# Modification of Polysulfone Ultrafiltration Membranes via Addition of Anionic Polyelectrolyte Based on Acrylamide and Sodium Acrylate to the Coagulation Bath to Improve Antifouling Performance in Water Treatment

**DOI:** 10.3390/membranes10100264

**Published:** 2020-09-28

**Authors:** Tatiana V. Plisko, Alexandr V. Bildyukevich, Katsiaryna S. Burts, Tatiana A. Hliavitskaya, Anastasia V. Penkova, Sergey S. Ermakov, Mathias Ulbricht

**Affiliations:** 1 Department of Analytical Chemistry, St. Petersburg State University, 7/9 Universitetskaya nab., 199034 St. Petersburg, Russia; a.penkova@spbu.ru (A.V.P.); s.ermakov@spbu.ru (S.S.E.); 2 Institute of Physical Organic Chemistry, National Academy of Sciences of Belarus, 13 Surganov str., 220072 Minsk, Belarus; uf@ifoch.bas-net.by (A.V.B.); katyaburt@gmail.com (K.S.B.); hlyavitskaya1706@gmail.com (T.A.H.); 3 Lehrstuhl für Technische Chemie II, and Center for Water and Environmental Research (ZWU), University of Duisburg-Essen, 45141 Essen, Germany; mathias.ulbricht@uni-essen.de

**Keywords:** membrane, polyelectrolyte, antifouling performance, ultrafiltration, water treatment

## Abstract

Surface modification of polysulfone ultrafiltration membranes was performed via addition of an anionic polymer flocculant based on acrylamide and sodium acrylate (PASA) to the coagulation bath upon membrane preparation by non-solvent induced phase separation (NIPS). The effect of PASA concentration in the coagulant at different coagulation bath temperatures on membrane formation time, membrane structure, surface roughness, hydrophilic-hydrophobic balance of the skin layer, surface charge, as well as separation and antifouling performance was studied. Scanning electron microscopy (SEM), atomic force microscopy (AFM), Fourier transform infrared (FTIR) spectroscopy, contact angle and zeta potential measurements were utilized for membrane characterization. Membrane barrier and antifouling properties were evaluated in ultrafiltration of model solutions containing human serum albumin and humic acids as well as with real surface water. PASA addition was found to affect the kinetics of phase separation leading to delayed demixing mechanism of phase separation due to the substantial increase of coagulant viscosity, which is proved by a large increase of membrane formation time. Denser and thicker skin layer is formed and formation of macrovoids in membrane matrix is suppressed. FTIR analysis confirms the immobilization of PASA macromolecules into the membrane skin layer, which yields improvement of hydrophilicity and change of zeta potential. Modified membrane demonstrated better separation and antifouling performance in the ultrafiltration of humic acid solution and surface water compared to the reference membrane.

## 1. Introduction

In recent years, consumption of fresh water has been increased due to the population growth and rapid industry development [1]. Ultrafiltration (UF) is a commonly used membrane process for surface water pretreatment and wastewater reclamation/reuse before reverse osmosis and nanofiltration [2]. Nowadays, UF is utilized in many real water treatment plants [2]. However, membrane fouling is known to be a severe unsolved problem which prevents sustainable membrane application and leads to the increased energy demand, flux decline, deterioration of separation performance and decrease of membrane lifetime [3,4]. This yields a significant increase of operation cost of water treatment and purification. It is widely accepted that there are four types of membrane fouling: colloidal fouling, biofouling, organic fouling, and inorganic fouling (scaling) [5]. Three main types of membrane foulants in water treatment are reported: (1) natural organic matter (NOM) where humic and fulvic substances are the main constituents; (2) algal organic matter composed of extracellular and intracellular macromolecules and cellular remains; and (3) wastewater effluent containing background (drinking water) NOM and soluble microbial products which are produced from biological wastewater treatment [5]. Several strategies have been applied to diminish effect of membrane fouling: feed water pretreatment (pre-coagulation, pre-oxidation) [6,7,8,9], tuning the membrane structure to decrease “membrane-foulant” interactions [10], development of different cleaning protocols (air sparging, backflushing and cleaning-in-place) [1,4] and adjusting membrane operation conditions (membrane module design, flow configuration and flow rate). Four main fouling mechanisms were discussed: blocking (plugging of the pore opening), standard blocking (clogging as a result of instantaneous adsorption of foulant on the pore wall), intermediate blocking (random precipitation on the membrane surface) and cake filtration (even growth of a cake or gel layer on the surface) [11]. However, real fouling process usually presents a combination of fouling mechanisms and has to be described by combined models [12].

Nature of membrane material, membrane structure (pore size, roughness of the selective layer) and physical-chemical properties of the selective layer (free surface energy, contact angle, zeta-potential) were found to significantly affect the adsorption of foulants on membrane surface as well as the structure and resistance of the formed gel layer during fouling [3,5]. It was shown that membranes with larger pore size were more subject to fouling [5]. It was reported that roughness has greater impact on fouling than hydrophobicity [5]. A greater (negative) charge was reported to reduce fouling in ultrafiltration [5]. Therefore, the main membrane modification strategies to reduce fouling involve enhancing hydrophilicity, decrease of surface roughness and tuning zeta potential of the selective layer.

Membrane modification techniques for hydrophilization and improvement of antifouling performance involve (1) chemical modification of membrane material, (2) blending with block-copolymers [13,14,15], hydrophilic polymers, oligomers [16,17,18,19] and nanoparticles [20], (3) surface segregation [21], (4) grafting [22,23,24], (5) surface coating [25,26], (6) nanoparticle immobilization on the selective layer surface [27] and (7) nanoparticle deposition [28].

Modification by polyelectrolyte is a promising approach for development of antifouling membranes due to polyelectrolyte’s high hydration ability, possibility to tune surface charge and versatility of chemistry, structure and modification techniques which can be implemented [29,30,31,32,33,34,35,36,37,38,39,40,41,42,43,44,45,46,47,48,49,50,51,52,53,54,55,56,57,58,59,60]. Moreover, modification by polyelectrolytes is a flexible instrument for design of thin film composite membranes for microfiltration [29], ultrafiltration (UF) [30,31,32,33,34,35,36], nanofiltration (NF) [37,38,39,40,41,42,43,44,45,46,47,48,49,50,51,52,53,54], reverse osmosis (RO) [55,56], forward osmosis [57,58] and pervaporation [59,60] as it allows creating ultrathin selective layers with controllable thickness via layer-by-layer (LBL) technique [29,33,34,35], coating [44,45] and grafting [30,31]. Coating and LBL assembly are the most frequently used techniques for membrane modification by polyelectrolytes due to relatively easy operation, flexibility, adjustable film thickness and good hydrophilicity compared to conventional chemical modification strategies (grafting). Grafting is usually a very time and labor-consuming, complex multistage technique which requires additional reagents and equipment [30,31]. Membrane modification by deposition of polyelectrolyte multilayers yields the improvement of antifouling performance for UF, NF and RO membranes [33,34,35], tailoring pH and ion-strength responsiveness [47] and adjustment of the retention of charged substances (proteins [35], multivalent metal ions [39,41,42,50], low molecular weight pharmaceuticals [46]) and viruses [29] due to a combination of associated pore narrowing effect and tuning surface charge. Moreover, polyelectrolyte multilayers can serve as defect-healing [61] and sacrificial layers [49,56] to remove adsorbed foulants from membrane surface. Both coating and LBL techniques can be followed by cross-linking [58] to enhance membrane stability and carried out in physical adsorption mode (dip-coating) [59,60] or dynamic mode [62,63,64] to decrease time of membrane formation. The main disadvantage of polyelectrolyte multilayer membranes is that their stability highly depends on pH and ionic strength of the feed [53]. Moreover, depending on the nature of polyelectrolyte pairs used and support membrane polyelectrolyte multilayer membrane can be unstable to oxidative cleaning (sodium hypochlorite or hydrogen peroxide) and physical cleaning (back flush) [53].

This paper presents a follow-up in depth investigation of the modification approach for design of antifouling ultrafiltration membranes via addition of polyelectrolyte to the coagulation bath proposed in our previous works [65,66]. It was established that additives to coagulation bath (salts [67,68,69,70], acids [71,72], bases [72,73], solvents [74,75], non-solvents [76]) during membrane preparation via non-solvent induced phase separation (NIPS) significantly influence the thermodynamics and kinetics of phase inversion. Additives to coagulation bath were found to adjust liquid-liquid demixing which leads to the substantial change of membrane structure and performance. For instance, adding solvent into the coagulation bath commonly was found to delay the demixing process which suppresses the formation of macrovoids and decreases membrane porosity resulting in the decline of permeability [75]. The influence of salt addition (KCl, NaCl, CaCl_2_) to the aqueous coagulation bath using polyvinylchloride-N-methyl-2-pyrrolidone (NMP) and polyvinylidene fluoride (PVDF)-NMP casting solutions with Pluronic F127 or polyethylene glycol (M_n_ = 12,000 g·mol^−1^) additives was studied [67,68,69]. Influence of NaCl, KCl and Na_2_SO_4_ addition to the aqueous coagulation bath for membranes prepared from polysulfone (PSF)-polyethylene glycol (PEG, M_n_ = 400 g·mol^−1^)-NMP casting solution was also investigated [70]. When polyelectrolyte is used as an additive to the casting solution, the membrane structure and performance are sensitive to the change of pH and ionic strength of the coagulation bath [71,72]. For instance, it was shown that the change of pH and ionic strength of the coagulation bath influences the self-assembly and conformation of amphiphilic triblock copolymer poly(N,N-dimethylamino-2-ethyl methacrylate)-block-polyethersulfone-block-poly(N, N-dimethylamino -2-ethyl methacrylate) (pDMAEMA-b-PES-b-pDMAEMA) which was used as an additive for the polyethersulfone (PES) casting solution in N,N-dimethylacetamide (DMA) for membrane preparation via NIPS [71]. Thus, coagulation bath composition influenced membrane structure, performance and responsiveness to external stimuli [71].

According to the literature review only a few studies were reported on the effect of addition of polyelectrolytes (polyacrylic acid (PAA) [77], polyethyleneimine (PEI) [78], copolymer of acrylamide and 2-acryloxyethyltrimethylammonium chloride (Praestol 859) [65,66] and hydrophilic polymer (PVP) [79] to the coagulation bath upon membrane preparation via NIPS. This approach presents facile, easy to perform, one-step method of immobilization of polyelectrolyte into the membrane structure without complex chemical reactions [65,66]. The method allows membrane hydrophilization, imparting charge of the selective layer as well as improvement of separation and antifouling membrane performance. Membrane modification via addition of copolymer of acrylamide and 2-acryloxyethyltrimethylammonium chloride (Praestol 859) to the coagulation bath upon PSF and PES membrane preparation via NIPS was reported [65,66]. FTIR studies of the membrane have shown that cationic polyelectrolyte macromolecules were immobilized into the membrane selective layer [65,66]. It was found that immobilization of Praestol 859 decreased water contact angle, increased surface roughness and changed zeta potential of the membrane selective layer. These changes were found to improve membrane stability to fouling toward NOM and human serum albumin [65,66]. Moreover, addition of Praestol 859 to the coagulation bath in case of PES membrane yields formation of macrovoid-free membrane matrix, increase of membrane permeability (2–6 times), improvement of fouling recovery ratio and cleaning efficiency. Rejection of hemicelluloses and lignin remained at the same level of 91.5–93% and 21–22% respectively [66].

A novel reaction enhanced surface segregation method was proposed for creating of separation layer with enhanced antifouling stability on the membrane surface [77]. PVP was added to the casting solution and acted as the surface segregation agent. Polyacrylic acid (PAA) was added to the coagulation bath. It was found that a crosslinked layer is formed due to the migration of PVP and PAA to the surface of the PVDF membrane during NIPS and hydrogen bond formation between PVP and PAA functional groups [77].

The novelty of the present study is that for the first time an anionic polyelectrolyte based on acrylamide and sodium acrylate (PASA) was added to the coagulation bath upon PSF membrane preparation via NIPS. Membrane modification by PASA addition to coagulation bath was previously not reported. It is worth mentioning that PASA is a widely used flocculant for water treatment, thus it is produced on a large scale and is not expensive. It is important from the point of view of availability and cost of membrane modification [80,81,82].

The aim of the study is to investigate the impact of the addition of anionic flocculant based on acrylamide and sodium acrylate to the coagulation bath on the structure, separation and antifouling performance of polysulfone ultrafiltration membranes.

## 2. Materials and Methods

### 2.1. Materials

**Figure 1:**
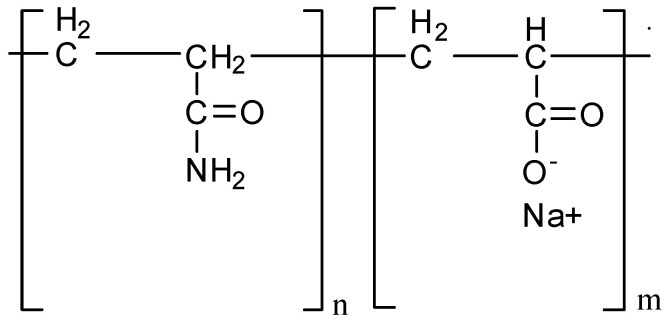
Chemical formulae of polymer flocculant based on acrylamide and sodium acrylate (PASA).

Polysulfone (PSF, Ultrason S 6010, M_n_ = 55,000 g∙mol^−1^, BASF, Ludwigshafen, Germany), polyethylene glycol (PEG, M_n_ = 400 g∙mol^−1^, BASF, Ludwigshafen, Germany) and N,N-dimethylacetamide (DMA, BASF, Ludwigshafen, Germany) were used as membrane material, pore-former and solvent, respectively. To prepare membranes via non-solvent induced phase separation (NIPS) tap water and aqueous solutions of the commercial flocculant Praestol 2540 (PASA, Ashland Inc., Covington, KY, USA) were used as a coagulant. PASA is an anionic flocculant, a copolymer of acrylamide and sodium acrylate (M_n_ = (10–14) × 10^6^ g∙mol^−1^, content of charged groups 40 mol. %). PASA finds applications as flocculant for water treatment [80,81,82]. The chemical formula of PASA is depicted in Figure 1.

Human serum albumin (HSA, M_w_ = 66,400 g∙mol^−1^, pI = 4.6; Sigma-Aldrich, St. Louis, MO, USA) was used as a model protein to evaluate membrane separation performance. For ultrafiltration experiments 0.5 wt % HSA solution in phosphate buffer with pH 7.0 was used as a feed.

0.001 wt % humic acid solution (HA) in tap water was used to evaluate the antifouling performance of the reference and modified membranes. The solution of HA was obtained from the fertilizer “hydrohumin” (Biochem, Belarus). “Hydrohumin” consists of the 50 wt % solution of HA in water.

### 2.2. Casting Solution Preparation

For casting solution preparation PSF, PEG and DMA were mixed for 4 h at 110 °C using an overhead stirrer at 700 rpm. Casting solution composition was 18 wt % PSF, 15 wt % PEG and 67 wt % DMA. Thereafter, the casting solution was left for 24 h for cooling down to room temperature (T = 22 °C) and degassing.

### 2.3. Preparation of PASA Solutions for Coagulation Bath

To prepare 0.05 wt %, 0.1 wt %, 0.2 wt % and 0.3 wt % PASA solutions the calculated quantity of anionic polyelectrolyte was added to 3 L of distilled water and mixed with overhead stirrer for 9 h at room temperature at 300 rpm. To remove gel particles, PASA aqueous solutions were filtered using a glass Schott filter (d_pore_ = 140 µm), a Bunsen flask, and a water jet pump before to application as a coagulation bath.

### 2.4. Measurements of the Viscosity

Dynamic viscosity of the PASA aqueous solutions at different temperatures (25, 40, 50, 60, 70 °C) was determined using Brookfield DV III Ultra rotary viscometer.

### 2.5. Precipitation Value of PASA Solutions

To evaluate the influence of PASA addition to the coagulant on the thermodynamics of the phase separation of PSF solution in DMA, precipitation values (PV) of 0.05–0.3 wt % PASA solutions were measured via cloud point titration technique. PV was determined as the quantity of the coagulant (g), necessary to induce the phase separation of 100 mL (1 dL) of 1 wt % PSF solution in DMA. The detailed procedure is described in [19]. Three different samples of polymer solution were titrated under constant stirring and the average PV was calculated. The accuracy of PV determination was 0.01 g. The cloud point was recorded if the solution remained turbid after 30 min of constant stirring.

### 2.6. Preparation of Membranes

Membranes were prepared by non-solvent induced phase separation (NIPS). In NIPS a one-phase casting solution separates into two phases upon the contact with coagulant (usually water): a solid, polymer-rich phase that forms the matrix of the membrane and a liquid, polymer-poor phase that forms the membrane pores. To prepare PSF flat-sheet membranes a casting solution (18 wt % PSF, 15 wt % PEG and 67 wt % DMA) at room temperature (T = 22 °C) was used. Rough glass substrate (average profile roughness, R_a_ = 1.4–1.6 μm) was utilized for membrane fabrication [65]. A doctor blade with a gap thickness of 250 μm was applied. The thickness of the prepared membranes was 50–60 µm. Tap water or PASA aqueous solutions of different concentrations (0.05 wt %, 0.1 wt %., 0.2 wt % and 0.3 wt %) at different temperatures (25, 40, 60 and 70 °C) were used as coagulation bath (Table 1). The prepared membranes were kept in distilled water for at least 24 h to remove traces of the DMA.

### 2.7. Membrane Formation Time

The membrane formation time (MFT) was determined as the time needed for as cast polymer film to precipitate and to separate spontaneously from the glass substrate during membrane formation via NIPS [65,66]. MFT was measured for different compositions of coagulant at T = 25 °C, including tap water and 0.05 wt %, 0.1 wt %, 0.2 wt % and 0.3 wt % PASA aqueous solutions.

### 2.8. Determination of Flux and Separation in Ultrafiltration

The method for determination of flux and rejection (R, %) is described in the previous work [19,65]. Ultrafiltration was carried out at trans-membrane pressure of 1 bar, room temperature and stirrer rotation speed 200 rpm. Pure water flux (PWF, L·m^−2^·h^−1^) and flux of 0.5 wt % HSA solution in phosphate buffer were measured. The ratio of optical densities at a wavelength of 280 nm of feed and permeate was calculated to determine rejection. For measurement of membrane separation performance at least five different membrane samples were tested and the average flux and rejection were calculated. The relative error was found to be less than 5% for flux measurements and less than 1% for rejection.

### 2.9. Study of Membrane Resistance to Fouling

To investigate membrane resistance to fouling, 0.001 wt % solution of humic acids (HA) in tap water was applied as a feed solution. According to the reported studies this HA concentration of the feed solution is typical for investigation of antifouling performance of PSF and PES membranes [83,84,85].

First, the ultrafiltration of distilled water was carried out at 1 bar for 30 min and pure water flux was measured. Thereafter, 0.001 wt % solution of HA in tap water was placed in ultrafiltration cell. The protocol for measurements was the following:

(1) HA solution ultrafiltration for 60 min with flux measurements every 15 min;
(2) distilled water ultrafiltration for 15 min (cleaning);
(3) determination of PWF of the washed membranes (JWF).

This protocol was repeated twice. Flux recovery ratio (FRR), reversible flux decline ratio (DR_r_), irreversible flux decline ratio (DR_ir_) and total flux decline ratio (DT) were calculated according to [3,85].

### 2.10. FTIR Studies

The composition of the membrane skin and bottom layers was studied by Fourier transform infrared spectroscopy (FTIR) using the spectrometer Nicolet Is50 (ThermoFisher Scientific, Waltham, MA, USA) in attenuated total reflectance mode (crystal material—diamond, angle of incidence 45°). Prior to the measurements membrane samples were dried at ambient conditions for 5 days.

### 2.11. Investigation of the Morphology

Scanning electron microscopy (SEM) and atomic force microscopy (AFM) were applied for membrane structure studies. To avoid pore contraction upon drying, membranes were put in 10 wt % glycerol aqueous solution for 1 h and dried at room temperature.

The membrane cross-sections were studied using SEM (instrument LEO 1420, LEO Electron Microscopy Inc., Thornwood, NY, USA) after gold layer sputtering (DSR1, Vaccoat, UK).

The structure of the skin layer was investigated using HT-206 atomic force microscope with standard silicon cantilevers with a spring constant of 3 N∙m^−1^ (MikroMash, Wetzlar, Germany).

### 2.12. Determination of Water Contact Angle

Water contact angle (θ, °) was measured by the captive bubble method using the LK-1 goniometer (Open Science, Krasnogorsk, Russia). The membrane was fixed in the cuvette with distilled water and air bubble of 2 µL was formed using a micro syringe. When the air bubble was moving from the bottom to the surface of the cuvette it attached spontaneously to the membrane surface (skin layer). The images of the system “membrane-air bubble” in water were taken by camera at magnification in 24 times and processed by image analysis software DropShape.

### 2.13. Zeta potential of the Skin Layer

Zeta potential of membrane skin layer was determined using electrokinetic analyzer SurPASS 2 (Anton Paar, Graz, Austria); the method is in detail reported in [65].

### 2.14. Analysis of Surface Water and Humic Acid Solutions

Surface water (Slepianski Channel, Minsk, Belarus) was used as a feed. Iron concentration was determined by an inductively coupled plasma atomic emission spectrometer (Vista PRO, Varian, Palo Alto, CA, USA). Total organic carbon was determined by Multi N/C UV HS TOC analyzer (Analytik Jena AG, Jena, Germany). Turbidity was measured by 2100 AN turbidimeter (HACH, Loveland, CO, USA). The optical density of feed surface water and permeate was determined using Metertech UV/VIS SP 8001 spectrophotometer (Metertech, Taipei, Taiwan) at a wavelength of 400 nm.

### 2.15. Surface Water Ultrafiltration

The procedure for surface water ultrafiltration is reported in detail in [65]. The ultrafiltration of distilled water was carried out at 1 bar for 30 min. Thereafter, surface water was filtered at P = 1 bar at room temperature in stirred mode for 60 min. The volume of permeate was measured every 30 s. The normalized flux (*J*_norm_) was calculated using the Equation (1):

(1) $$J_{\mathrm{norm}}=\frac{J}{J_{0}}$$

where *J*_0_ (L·m^−2^·h^−1^) is the flux of surface water in the beginning of filtration and *J* (L·m^−2^·h^−1^) is the flux of surface water measured at a certain time of filtration.

## 3. Results

**Figure 2:**
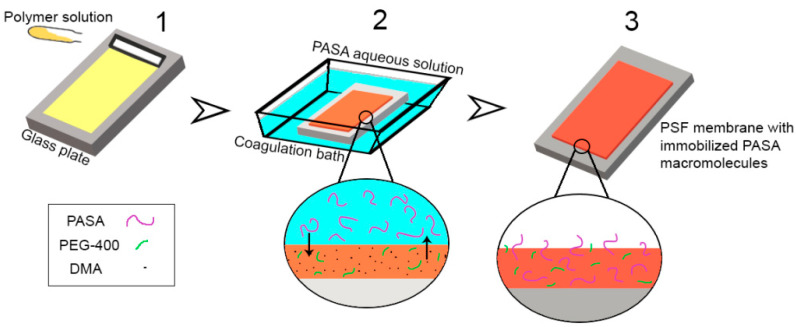
Schematic representation of the formation of polysulfone (PSF) flat-sheet membranes via NIPS using PASA aqueous solution as a coagulation bath: (**1**)—casting of the PSF solution on the glass plate using doctor blade; (**2**)—immersion of the cast polymer film in the coagulation bath, membrane structure is formed due to the solvent-non-solvent exchange (PEG and DMA diffuse out of the polymer film and PASA aqueous solution diffuses into the polymer film); (**3**)—PSF membrane with immobilized PASA macromolecules is obtained.

It was found that addition of cationic polyelectrolyte (copolymer of acrylamide and 2-acryloxyethyltrimethylammonium chloride) (Praestol 859) [65,66] and hydrophilic polymer (polyvinylpyrrolidone) [79] to the coagulation bath or bore fluid results in the immobilization of polymer additive into the membrane. This occurs due to the diffusion of macromolecules from bulk of coagulant liquid to the as cast polymer film and subsequent anchorage in membrane matrix when membrane polymer solidifies due to the contact with coagulant. It results in the changing composition and, therefore, hydrophilization and change of zeta potential of the membrane skin layer. It was expected that the same mechanism would work for PASA as well (Figure 2).

### 3.1. Effect of PASA Additive on Precipitation Value of PSF Solution in DMA

It is commonly known that addition of a polymer to the coagulant can affect both kinetics and thermodynamics of NIPS. However, similar to the case of the addition of Praestol 859 a significant influence of PASA addition on the precipitation value (PV) determined by cloud point titration technique of 1 wt % PSF solution in DMA was not detected [65]. The PV of 1 wt % PSF in DMA for both distilled water and 0.05–0.5 wt % PASA aqueous solutions was measured to be 4 g·dL^−1^. Based on the data obtained the change of membrane structure should be attributed to the change of the viscosity of the coagulation bath which slows down the rate “non-solvent-solvent exchange” in NIPS, similar to what had been observed with the other polyelectrolyte before [65].

### 3.2. Effect of PASA Concentration and Temperature on Coagulation Bath Viscosity

**Figure 3:**
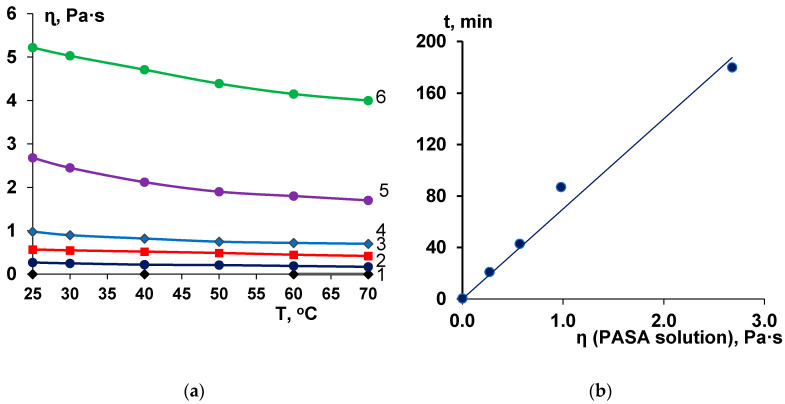
Dependence of the dynamic viscosity of the aqueous solutions of PASA at different concentrations (in wt %: 1—0; 2—0.05; 3—0.1; 4—0.2; 5—0.3; 6—0.5) on temperature (**a**) and correlation of membrane formation time at T = 25 ××°C with viscosity of PASA solutions (**b**).

Viscosity of the casting solution has an important influence on membrane structure formation. The viscosity increase can lead to the delayed demixing mechanism and formation of macrovoid-free, spongy porous structure [86,87]. However, the effect of coagulant viscosity on membrane structure formation via NIPS was studied only in few works [65,66,70]. It was revealed that aqueous solutions of anionic polyelectrolyte PASA feature very high viscosity at low concentrations due to very high molecular weight (Figure 3a). The increase of concentration was found to yield a substantial increase of PASA solution dynamic viscosity (Figure 3a). For example, when PASA concentration increases from 0.05 wt % to 0.5 wt % the dynamic viscosity increases from 0.27 Pa·s to 5.22 Pa·s (19.1 times) (Figure 3a).

It was revealed that PASA aqueous solutions at all studied concentrations demonstrate slight sensitivity to temperature (Figure 3a). When the temperature increases from 25 °C to 70 °C the dynamic viscosity decreases 1.31–1.59 times depending on PASA concentration in the solution which is even less than the change of viscosity of water with the same temperature increase (2.22 times). For instance, when temperature increases from 25 °C to 70 °C the viscosity of 0.5 wt % aqueous PASA solution decreases from 5.22 to 4.0 Pa·s (in 1.31 times).

The results obtained indicate that dynamic viscosity of anionic polyelectrolyte PASA solutions substantially depends on even slight concentration change. The viscosity was revealed to depend on temperature to much lower extent.

### 3.3. Effect of PASA Concentration on Membrane Formation Time in NIPS

A strong impact of the PASA solution viscosity on the membrane formation time was found (Figure 3b). A linear dependence of membrane formation time on coagulant viscosity was revealed (Figure 3b). When coagulation bath viscosity increases the solvent-non-solvent exchange rate in NIPS decreases, leading to the large rise of membrane formation time. For instance, the increase of coagulant viscosity from 0.00089 Pa·s (water) to 0.27 Pa·s (0.05 wt % PASA) results in the increase of membrane formation time by 42 times (from 0.5 min to 21 min). When coagulation bath viscosity increases from 0.27 Pa·s (0.05 wt % PASA) to 2.68 Pa·s (0.3 wt % PASA) membrane formation time increases by 360 times.

It can be concluded that PASA addition to the coagulation bath strongly affects the kinetics of NIPS due to the substantial viscosity increase, however, the thermodynamics is not influenced. It is proved by the similar PV values of PSF solution in DMA for PASA aqueous solutions and water.

### 3.4. Effect of PASA Addition to the Coagulation Bath on Membrane Composition

**Figure 4:**
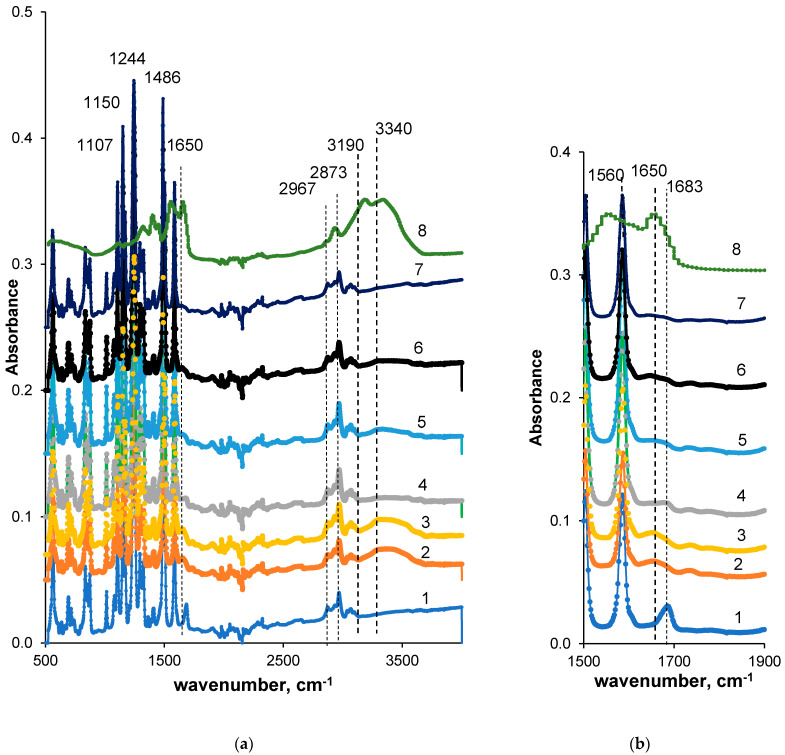
FTIR spectrum (**a**) and fragment of the spectrum (**b**) of the PSF membrane skin (2–5) and bottom (6, 7) layer; PASA concentration in the coagulant, wt %: 1—0, T = 60 °C (A60); 2—0.2, T = 60 °C (A-0.2-60); 3—0.2, T = 25 °C (B-0.2-25); 4—0.3, T = 60 °C (A-0.3-60); 5—0.3, T = 25 °C (A-0.3-25); 6—0.2, T = 60 °C, bottom layer (A-0.2-60); 7—0.2, T = 25 °C (A-0.2-25), bottom layer; 8—PASA powder.

To confirm the immobilization of PASA macromolecules from the coagulation bath the FTIR spectra were recorded for both skin and bottom layer of membranes prepared using different PASA concentrations at coagulation bath temperatures 25 and 60 °C (Figure 4).

It was revealed that both reference PSF and PSF/PASA membranes feature a number of characteristic peaks typical of vibrations of the groups of membrane material (PSF) (Figure 4). It indicates the membrane matrix of modified membrane presents a blend of PSF and PASA. Stretching vibrations of the SO_2_ group yield the peak at 1294 cm^−1^ (1–7 in Figure 4). The peak at 1150 cm^−1^ is assigned to the –C–SO_2_–C– symmetric stretching vibrations [79,88]. Absorbance peaks at 1107, 1486, 1585 cm^−1^ are ascribed to the aromatic ring of PSF [79,88]. The absorbance vibration at 1244 cm^−1^ is assigned to ether bond of PSF connected to aromatic ring [79,88,89]. Stretching vibrations of the methyl groups are found at 2873 and 2967 cm^−1^ (1–5 in Figure 4). The vibration band at 1683 cm^−1^ for the reference membrane prepared at coagulation bath temperature 60 °C (A60) refers to the contributions from C=O and N–C stretching vibrations from DMA that remained in membrane matrix (1 in Figure 4). However, traces of PEG also can remain in membrane matrix and contribute to the absorbance peak at 1107 cm^−1^ (C–O–C– stretching vibrations) overlapping with PSF peaks.

Analysis of PASA FTIR spectrum reveals the broad band in the range of 3150–3500 cm^−1^ with two maxima at 3340 and 3190 cm^−1^ (8 in Figure 4). The maximum at 3340 cm^−1^ corresponds to the vibrations of –NH_2_ groups of PASA acrylamide units involved in hydrogen bond formation. The latter maximum at 3190 cm^−1^ is due to hydroxyl groups of acrylic acid units (not in Na^+^ salt form). The peak at 2930 cm^−1^ is attributed to the stretching vibrations of single carbon-hydrogen bond [90]. The broad band with the maximum at 1650 cm^−1^ corresponds to overlapping of carbonyl group vibrations from amide and carboxylate groups. Peak at 1400 cm^−1^ is assigned to NH in plane bending of amide II, peak at 1560 cm^−1^ to NH deformation vibration, peak at 1320 cm^−1^ to stretching vibrations of carboxylate groups and peak at 1100 cm^−1^ is attributed to C–O stretching vibrations.

When PASA is added to coagulant a small broad peak at 1650 cm^−1^ appears in membrane FTIR spectrum which is the result of overlapping of vibrations of C=O groups (in COO– and CONH_2_ groups of PASA as well as CONH_2_ groups of remaining DMA). Moreover, a broad peak with the maximum at 3340 cm^−1^ is observed in FTIR spectrum of modified membranes (NH_2_ groups from PASA). These changes of the membrane spectra compared to the spectra of reference membrane prove the incorporation of PASA macromolecules to membrane matrix.

Comparison of spectra A60, A-0.2-60, A-0.3-60 membranes (1, 2, 4 in Figure 4) allows concluding that more PASA molecules were immobilized when 0.2 wt % PASA was added to coagulation bath. This is due to the significantly lower viscosity of 0.2 wt % PASA solution compared to 0.3 wt % solution (3, 4 in Figure 3). High viscosity of the coagulant in case of 0.3 wt % PASA restricts the diffusion of the macromolecules into the polymer film during membrane formation; thus, less PASA macromolecules have the opportunity to be incorporated in the membrane matrix. A similar trend is observed for spectra of A-0.2-25 and A-0.3-25 membranes prepared at coagulation bath temperature 25 °C (3, 5 in Figure 4). It was found that coagulation bath temperature (25 and 60 °C) does not affect the spectra of PSF/PASA membranes both for skin and bottom layers (2–7 in Figure 4). However, membrane selective layer was revealed to be modified to higher extent compared to the membrane bottom layer (compare 2 and 6 with 3 and 7 in Figure 4). This occurred due to extremely high viscosity of PASA solutions used as a coagulant in NIPS which restricts in-diffusion of coagulant into the as-cast polymer film and thus dramatically increases membrane formation time (Figure 4a,b).

### 3.5. Effect of PASA Addition to the Coagulant on Membrane Morphology

#### 3.5.1. SEM Investigations

**Figure 5:**
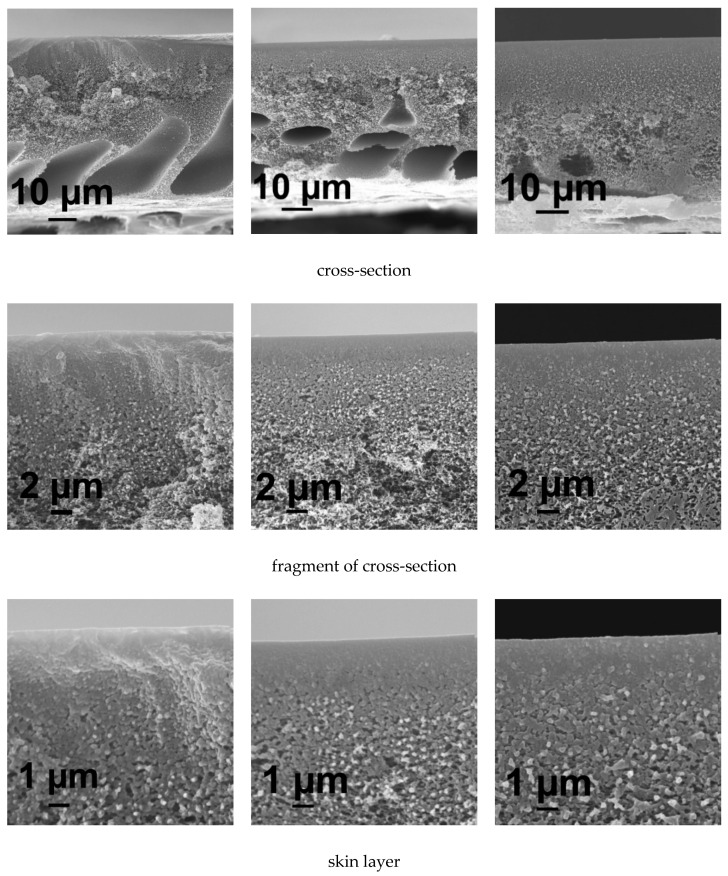

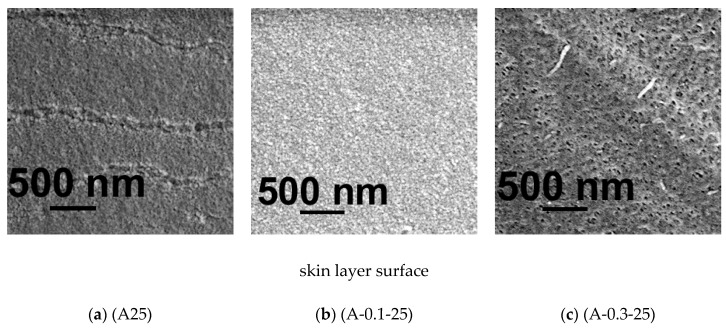
SEM micrographs of the membrane cross-section, enlarged fragment of the cross-section, skin layer and surface of the skin layer, coagulation bath, T = 25 °C, PASA concentration in the coagulant, wt %: (**a**) 0; (**b**) 0.1; (**c**) 0.3.

It was found that the reference membrane A25 prepared using water at 25 °C as a coagulant features anisotropic structure with wide and thick elongated macrovoids located near the membrane bottom layer (Figure 5a). The porous membrane matrix is formed of polymer globules. The size of pores and porosity of membrane matrix gradually increases from the top to the bottom of membrane in the cross-section (Figure 5a). When 0.1 wt % PASA is added to the coagulation bath the following changes of membrane structure take place: macrovoids become significantly smaller, their shape becomes flattened oval, the structure in the vicinity of the skin layer becomes denser compared to the reference A25 membrane (Figure 5a,b). Further increase of PASA concentration up to 0.3 wt % in the coagulant results in the practically macrovoid-free membrane structure formation (Figure 5c). Membrane matrix was found to become significantly denser and less porous compared to the A25 and A-0.1-25 membranes (Figure 5c). Bigger polymer globules are observed in the skin layer as well as in the membrane matrix of A-0.3-25 membrane (Figure 5c). A-0.3-25 membrane skin layer was revealed to become denser and its porosity substantially decreases (Figure 5c).

The abovementioned changes of membrane structure with PASA addition to the coagulant and increase of its concentration are attributed to the decrease of “solvent-non-solvent exchange” rate due to significant viscosity increase of the coagulant which yields the delayed demixing mechanism of NIPS (Figure 3a,b). It is commonly known that delayed demixing mechanism of phase separation suppresses macrovoid formation in membrane matrix [19,86,87].

The pore size on the surface of the skin layer was found to increase with the increase of PASA concentration in the coagulant especially when PASA concentration reaches 0.3 wt % (Figure 5).

**Figure 6:**
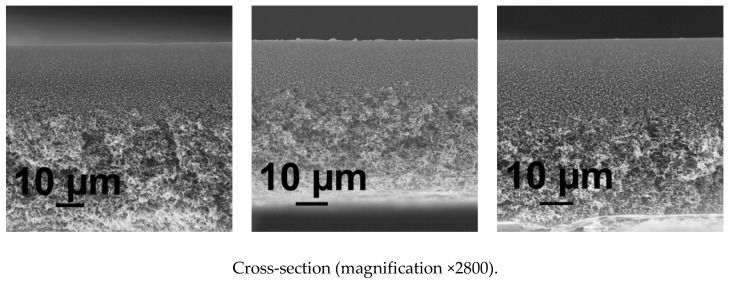

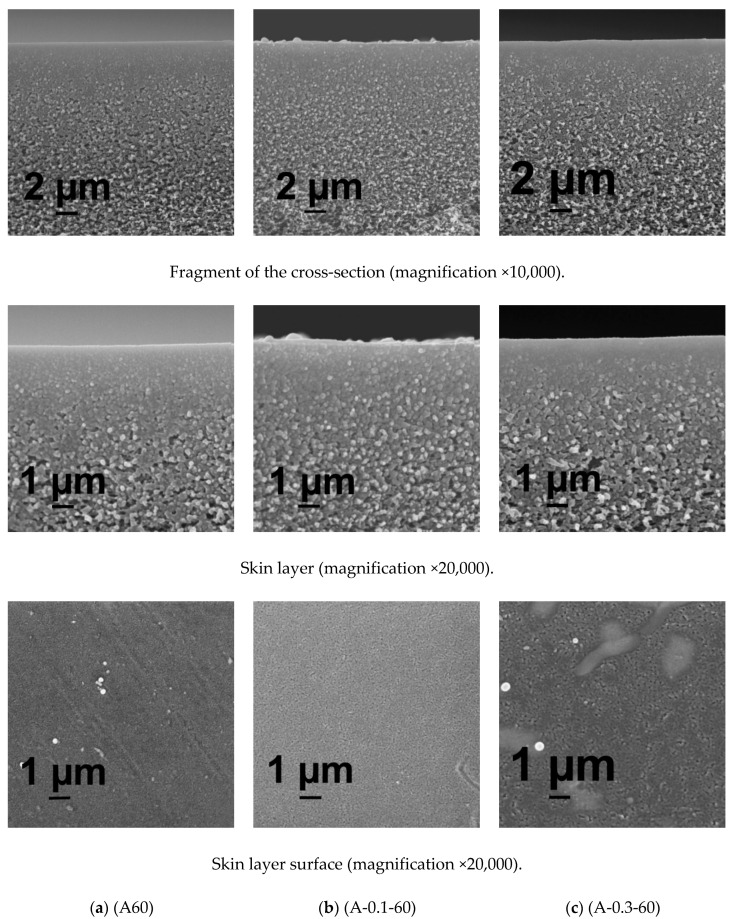
SEM micrographs of the membrane cross-section and enlarged fragment of cross-section, coagulation bath, T = 60 °C, PASA concentration in the coagulant, wt %: (**a**) 0; (**b**) 0.1; (**c**) 0.3.

It was found that the increase of coagulation bath temperature up to 60 °C leads to the formation of sponge-like membrane structure free of macrovoids, both in case of using water and PASA aqueous solutions as a coagulant (Figure 6). Macrovoids disappear due to the enhanced tolerance of the casting solution toward the coagulant at higher temperature (60 °C) when a thin cast polymer film is immersed in hot coagulation bath [87]. This leads to the delayed demixing in NIPS. Moreover, another important factor is the improvement of the homogeneity of PASA solution with the increase of temperature since PASA solubility in water is better at elevated temperatures.

However, at high coagulation bath temperature both the viscosity of polymer solution and coagulant significantly decreases (cf. Figure 3a) which leads to the increase of “solvent-non-solvent” exchange rate in NIPS. Obviously, the impact of this factor is much lower compared to the two above mentioned reasons (and this is in line with the lower influence of temperature change onto viscosity compared to that of a concentration change).

The morphology of the skin layer for membranes obtained at coagulation bath temperature 60 °C is more uniform and the thickness of the skin layer decreases compared to the membranes obtained at 25 °C (Figure 5 and Figure 6). It was found that when PASA content in the coagulant increases the thickness and density of the skin layer increase for membranes prepared at coagulation bath temperature 60 °C (Figure 6). It was observed that the size of polymer globules in the vicinity of the skin layer increases as well (Figure 6).

Consequently, pore size on the surface of the selective layer was found to substantially increase with the increase of PASA concentration at T = 60 °C (Figure 6)

#### 3.5.2. AFM Investigations

**Figure 7:**
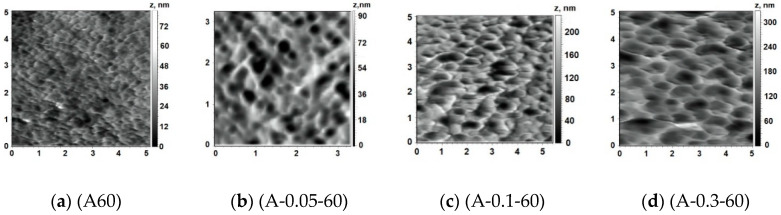
AFM images of the surface of the skin layer, PASA concentration in the coagulant, (T = 60 °C), wt %: (**a**) 0; (**b**) 0.05; (**c**) 0.1; (**d**) 0.3.

**Figure 8:**
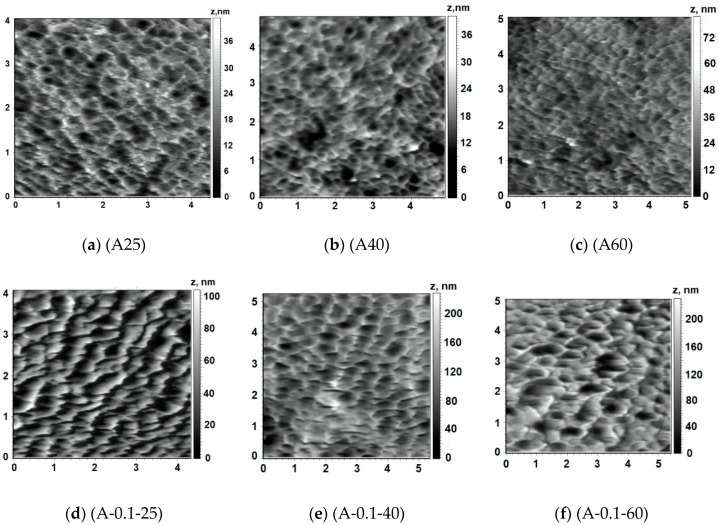
AFM images of the skin layer surface of PSF membranes, PASA concentration in the coagulant: (**a**–**c**) 0; (**d**–**f**) 0.1. Coagulation bath temperature, °C: (**a**,**d**) 25; (**b**,**e**) 40; (**c**,**f**) 60.

Structure of the surface of membrane skin layer is known to play the most important role in the “solute-membrane surface” interactions during ultrafiltration and, thus, determines membrane separation performance and antifouling resistance. The surface of the skin layer was found to demonstrate nodular structure, which consists of valleys with well-defined edges. Reference PSF membrane has very smooth surface regardless the temperature of coagulation bath during NIPS (Figure 7a and Figure 8a–c). It was revealed that PASA addition to the coagulation bath leads to the significant increase of the size of the valleys on the surface of the skin layer which results in the substantial increase of the surface roughness parameters (root-mean-squared surface roughness (R_q_) and average surface roughness (R_a_)) for membranes prepared at coagulation bath temperature 60 °C (Figure 7, Table 2). With the PASA content increase edges of the valleys become sharper, thicker and higher compared to the reference membrane (Figure 7). This is assigned to the slower solvent-non-solvent exchange rate due to higher viscosity of the coagulant which leads to the formation of bigger polymer nodules. Bigger polymer nodules generate larger and deeper valleys on the skin layer surface upon membrane formation via NIPS. This trend is in a good accordance with the increase of pore size of the skin layer surface with increase of PASA concentration revealed by SEM investigations (Figure 6).

Addition of 0.1 wt % PASA to the coagulant at coagulation bath temperature 25 °C was found to yield the formation of the membrane structure with larger valleys and sharper edges compared to the A25 membrane, however, surface roughness parameters did not significantly increase (Figure 8a,d; Table 2). This observation corresponds well to the increase of the pore size of the skin layer surface with the addition of 0.1 wt % PASA demonstrated by SEM (Figure 5a,b).

It was found that with the addition of 0.05 wt % PASA to the coagulant, R_a_ and R_q_ increase from 4.7 and 6.1 nm for the reference membrane up to 11.2 and 14.1 nm for A-0.05-60 membrane. Further increase of PASA concentration up to 0.1 wt % and 0.3 wt % leads to a substantial further increase of surface roughness parameters with the maximum values at 0.1 wt % PASA (Table 2).

##### Influence of Coagulation Bath Temperature

Two different patterns of the influence of coagulation bath temperature on the structure of the skin layer surface were revealed. When water is used as a coagulation bath the size of the valleys was found to decrease with the increase of temperature from 25 °C up to 40 and 60 °C.

Surface roughness parameters were found practically not to change with the temperature increase for the reference membrane (Table 2). However, A25 membrane surface demonstrates edges of the valleys which are composed of polymer globules. With the temperature increase these globules disappear from the edges (Figure 8a–c).

Another behavior with the increase of coagulation bath temperature was observed for membranes, prepared using 0.1 wt % PASA solution as a coagulant. The surface roughness parameters were revealed to increase sharply with the increase of temperature which is due to the increase of solvent-non-solvent exchange rate since the viscosity of coagulation bath decreases (Figure 8d–f, Table 2, Figure 3a).

### 3.6. Effect of PASA Addition to the Coagulant on Water Contact Angle of Membrane Skin Layer

**Figure 9:**
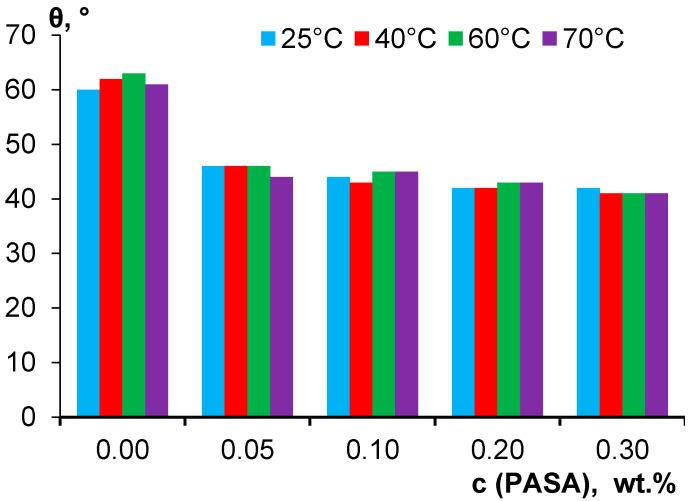
Water contact angle (θ) vs. PASA concentration in the coagulation bath at different coagulation bath temperatures.

Immobilization of hydrophilic PASA macromolecules was revealed to efficiently decrease water contact angle of membrane skin layer (Figure 9). This is attributed to the presence of highly hydrophilic carboxylate and amide groups due to PASA immobilization on the skin layer surface which was proved by FTIR (Figure 4). The water contact angle of reference membrane was determined to be 60–63° depending on coagulation bath temperature (Figure 9). The introduction of 0.05 wt % PASA to the coagulant yields the substantial decrease of water contact angle down to 44–46°. At 0.1 wt % PASA contact angle slightly decreases down to 43–45°, at 0.2 wt % - to 42–43° and at 0.3 wt %—to 41–42°. It should be noted that increase of PASA concentration from 0.05 wt % to 0.3 wt % does not yield the significant contact angle decrease which may be due to the substantial increase of surface roughness parameters discussed in the previous section (Figure 9, Table 2) [91]. According to FTIR spectra studies, coagulation bath temperature does not influence the intensity of the peaks of immobilized hydrophilic NH_2_– and carbonyl groups on the skin layer surface. This maybe the reason why coagulation bath temperature was found to practically not affect water contact angle, regardless the increase of surface roughness parameters (Figure 9, Table 2).

### 3.7. Effect of PASA Addition to the Coagulant on Zeta Potential of Membrane Skin Layer

Zeta potential of the skin layer surface of the reference, A25 and A-0.3-25 membranes at different pH was determined and compared (Table 3). Zeta potential of the skin layer is the important characteristic, which affects membrane separation and antifouling performance [92]. At pH = 3.2 both A25 and A-0.3-25 membrane demonstrate positive values of zeta potential. However, zeta potential of modified membrane was found to be 3.5 times higher compared to the zeta potential of the reference membrane, which is due to the protonation of sodium acrylate units of PASA incorporated into the membrane skin layer. A pH value of 4.0 is the isoelectric point of the membrane surface both for A25 and modified A-0.3-25 membrane in this particular ionic system (Table 3). With the further increase of pH, the absolute value of the negative zeta potential of the skin layer surface of both reference and modified membrane increases. At pH = 4.6 A25 membrane was found to demonstrate more negative zeta potential compared to the A-0.3-25 membrane. For pristine A25 membrane this reflects the higher ionic adsorption potential of anions resulting in preferential anionic adsorption due to their weaker hydration. Specific ionic adsorption is the only process possible for surface charge formation of the pristine A25 membrane as PSF has no dissociable functional groups [92]. In the case of unmodified membrane negative zeta potential is attributed to the adsorption of anions of electrolyte (Cl^−^) and hydroxyl ions (OH^−^) at pH = 4.6. In the case of modified A-0.3-25 besides specific ionic adsorption the dissociation of the –COOH group plays an important role in the formation of the surface charge. Both processes are competitive [92]. However, surface of the skin layer of A-0.3-25 membrane at pH = 4.6 has the regions, which consist of PASA macromolecules with protonated acrylate units that lead to a decrease of the absolute value of negative zeta potential. At pH 7.0 and 9.5 A-0.3-25 membrane features more negative zeta potential of the skin layer surface compared to the reference membrane due to the dissociation of the PASA acrylic acid units (yielding –COO– on the surface). It is supposed that transport properties of the pristine A25 membrane will not be influenced by the change of the feed solution pH. It is due to the fact that zeta potential change is caused by the adsorption of anions. On the contrary, in the case of modified A-0.3-25 membrane increase of pH leads to the ionization of carboxylic groups. It results in the change of macromolecule conformation of PASA from coiled to unfolded one due to electrostatic repulsion. It is expected to cause the decrease of the pore size which will yield the decrease of membrane permeability [93].

### 3.8. Membrane Barrier Properties

**Figure 10:**
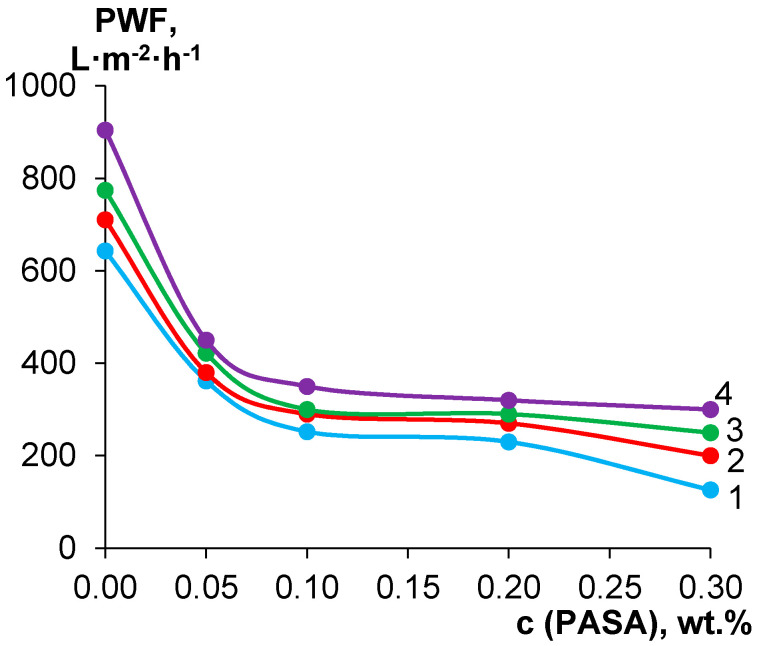
Dependence of pure water flux (PWF; measured at trans-membrane pressure of 1 bar) on PASA concentration in the coagulation bath at different coagulation bath temperatures, T, °C: 1—25; 2—40; 3—60; 4—70.

It was found that pure water flux significantly decreases when PASA had been added to the coagulation bath at all studied coagulation bath temperatures (Figure 10). This is due to the formation of denser and thicker skin layer upon increase of PASA content, which was demonstrated for both coagulation bath temperatures, 25 and 60 °C (Figure 5 and Figure 6). However, the pore size of the skin layer was found to increase with the increase of the PASA concentration in the coagulation bath both for coagulation bath temperatures 25 and 60 °C, which seems to contradict the trend of pure water flux decrease (Figure 5 and Figure 6). It is worth noting that the pore can change its shape and size across the thickness of the skin layer, and this cannot be detected by SEM investigations. In fact, SEM images of the skin layer surface are not informative enough to make conclusions about the correlation between membrane structure and performance. Moreover, not only pore size affects membrane permeability but also the porosity of the skin layer which also can change across skin layer thickness.

For instance, when 0.05 wt % PASA was added to the coagulant pure water flux decreases from 643.2 L·m^−2^·h^−1^ for the reference A25 membrane down to 362 L·m^−2^·h^−1^ for A-25-0.05 membrane (by 1.78 times). Further increase of PASA concentration up to 0.3 wt % yields the pure water flux decrease down to 126 L·m^−2^·h^−1^ which is 5.1 times lower compared to the pure water flux of the reference A25 membrane. Increase of coagulation bath temperature was found to increase permeability for both reference and modified membranes for all PASA concentrations in the coagulant; this is due to the formation of more uniform and thin skin layer (Figure 5 and Figure 6).

**Figure 11:**
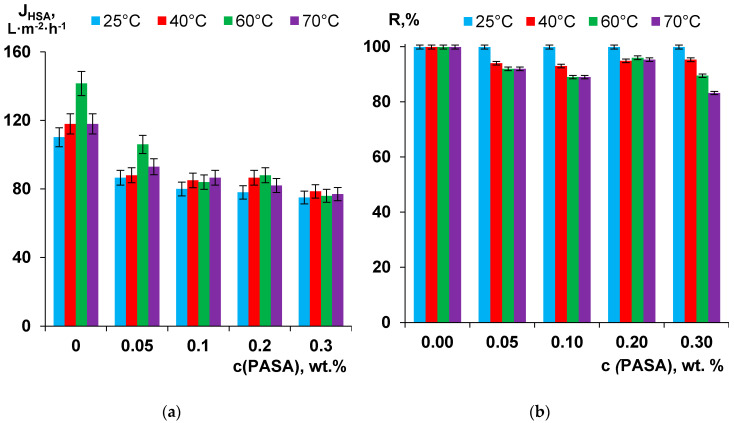
Human serum albumin (HSA) solution flux (J_HSA_; measured at trans-membrane pressure of 1 bar) (**a**) and HSA rejection (R) (**b**) of PSF membranes prepared at different PASA concentration in the coagulant at different temperatures.

The dependence of membrane separation performance on PASA concentration in the coagulant at different coagulation bath temperatures was studied using 0.5 wt % HSA solution at pH = 7.0 (Figure 11a). The trend of HSA solution flux decrease is similar to the trend for pure water flux decrease with the increase of PASA concentration in the coagulant. However, this decrease is not as big as in the case of pure water flux. For instance, the flux of A-0.3-25 membrane is only 1.47 times lower compared to the flux of the reference A25 membrane (Figure 11a). The effect of the coagulation bath temperature on the HSA solution flux was revealed to be more pronounced for the reference membrane prepared using water as a coagulant compared to PSF/PASA membranes. For reference membrane and membranes prepared using 0.05 wt % and 0.2 wt % PASA content in the coagulant maximum flux is observed after preparation at T = 60 °C. For membranes prepared using 0.1 wt % and 0.3 wt % PASA aqueous solutions as a coagulant slightly higher flux was determined at the coagulation bath temperature 70°C. However, it is worth nothing that the influence of coagulation bath temperature upon membrane preparation on HSA solution flux of PSF/PASA membranes is rather small (Figure 11a).

It was revealed that rejection of HSA is above 99.9% for membranes prepared using water as a coagulant at all studied coagulation bath temperatures (Figure 11b). It was found that rejection remains unchanged (99.9%) for membranes using PASA solutions at all concentrations at coagulation bath temperature 25 °C (Figure 11b). However, the increase of coagulation bath temperature up to 40–70 °C leads to the decrease of rejection when PASA is added to the coagulation bath. The minimal rejection of 89.5% and 83.2% is observed for A-0.3-60 and A-0.3-70 membranes respectively. There are two factors related to membrane properties which affect the rejection of HSA: pore size and zeta potential of the skin layer. According to SEM studies, pore size of the skin layer was found to increase with the increase of PASA concentration in the coagulation bath both for coagulation bath temperatures 25 and 60 °C (Figure 5 and Figure 6). It was demonstrated that modified membrane feature higher negative zeta potential of the skin layer which presumably have to increase the rejection of negatively charged HSA macromolecules at pH = 7 (Table 3). It can be assumed that in the case of PSF/PASA membranes prepared at coagulation bath temperature 25 °C the increase of pore size of the skin layer is countervailed by the increase of the absolute value of the negative zeta potential which results in the maintaining of high HSA rejection (99.9%) at all studied PASA concentrations (Figure 11b). However, at coagulation bath temperatures 40–70 °C pore size of the skin layer is higher and the degree of such compensation is lower yielding the decrease of HSA rejection (Figure 6 and Figure 11b).

### 3.9. Separation and Antifouling Performance in Ultrafiltration of Humic Acids Model Solution and Surface Water

**Figure 12:**
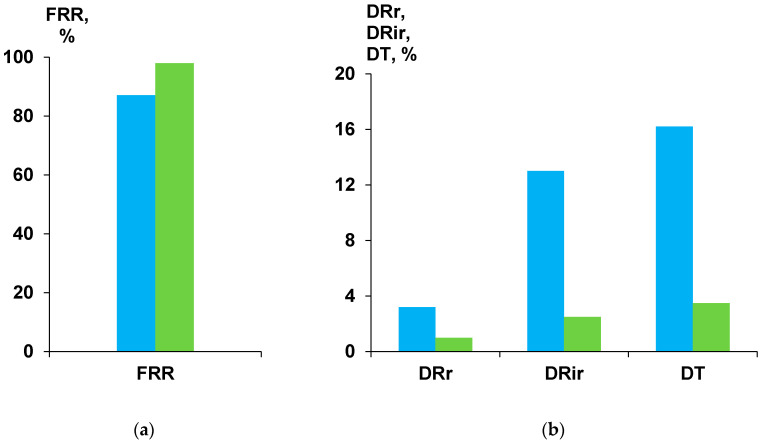
Fouling parameters (**a**) FRR and (**b**) DRr, DRir, DT in the process of 0.001% humic acid solution ultrafiltration using A25 (blue) and A-0.3-25 (green) membranes.

The effect of the PASA addition to the coagulation bath on the membrane antifouling performance was studied in the process of ultrafiltration of model solutions, i.e., 0.001 wt % of humic acids (HA) in tap water. The performance of reference A25 and modified A-0.3-25 membrane were compared in terms of antifouling parameters (flux recovery ratio (FRR), reversible flux decline ratio (DRr), irreversible flux decline ratio (DRir) and total flux decline ratio (DT)) (Figure 12). The HA solution flux in the beginning of the ultrafiltration was 390 L·m^−2^·h^−1^ for the reference A25 membrane and 480 L·m^−2^·h^−1^ for A-0.3-25 membrane (at 1 bar). After 60 min of ultrafiltration HA solution flux decreased to 312 L·m^−2^·h^−1^ for the reference membrane and to 468 L·m^−2^·h^−1^ for the modified A-0.3-25 membrane. The characteristics of the feed HA solution and permeate are presented in Table 4. Both A25 and A-0.3-25 membranes are characterized by efficient removal of color, organic carbon and iron. It was found that the feed HA solution has pH = 8.4. It is known that humic acids are characterized by the negative charge at pH = 8.4 [94]. The surface of the skin layer of A25 and A-0.3-25 membranes was also revealed to demonstrate negative charge being higher by absolute value for the modified membrane (Table 3). 0.1 wt % PASA addition to the coagulation bath yields the significant improvement of membrane antifouling performance: FRR increases from 87.1% for the reference membrane up to 98% for A-0.3-25 membrane. DT, DRr and DRir decreased from 16.2%, 3.2% and 13% for A-25 membrane down to 3.5%, 1% and 2.5%, respectively, for A-0.3-25 membrane (Figure 12). This efficient improvement of antifouling performance is due to the significant hydrophilization of the surface of skin layer and increasing of absolute value of negative zeta-potential compared to the reference membrane (Figure 9, Table 3). Immobilization of hydrophilic groups on the surface of the membrane skin layer upon PASA addition to the coagulation bath promotes the formation of thin water layer on the membrane surface in aqueous media which prevents the adsorption of foulants. Higher negative zeta-potential of the skin layer surface of modified membrane is responsible of the strong repulsion of negatively charged HA molecules from the membrane surface which also decreases the degree of membrane fouling.

**Figure 13:**
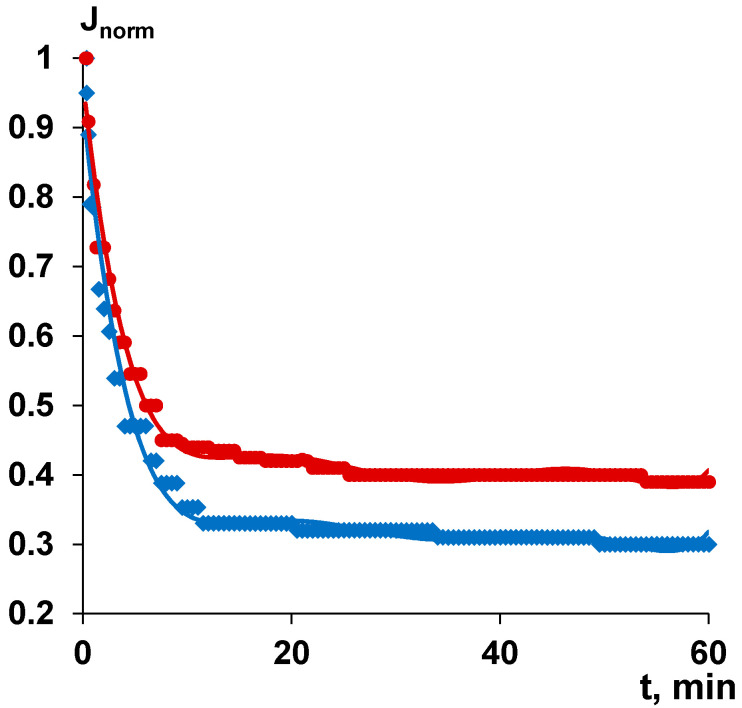
The dependence of normalized flux (J/J_0_) of surface water during ultrafiltration of reference (1—A60) and modified (2—A-0.3-60) membranes.

Membrane modification using PASA was revealed to improve separation and antifouling performance in surface water ultrafiltration (Figure 13). The flux of surface water usually sharply decreases with time due to membrane fouling by natural organic matter (NOM), colloid particles and microorganisms. The initial flux of surface water was 567 L·m^−2^·h^−1^ for the reference membrane A60 and 175 L·m^−2^·h^−1^ for modified A-0.3-60 membrane. The membranes prepared at the coagulation bath temperature 60 °C were selected for surface water ultrafiltration studies due to higher permeability. It is worth noting that the experiments on surface water ultrafiltration were carried out at the same trans-membrane pressure (1 bar) and not at the same initial flux, thus, the modified membrane (starting with higher flux) has been exposed to stronger fouling. It was found that the flux of both the reference and modified membrane dramatically decreases in first 10 min of ultrafiltration due to fouling and remains only 35% (198 L·m^−2^·h^−1^) for the A60 membrane and 44% (77 L·m^−2^·h^−1^) for modified A-0.3-60 membrane (Figure 13). Thereafter, flux decrease is much more smooth reaching 170 L·m^−2^·h^−1^ for the reference A60 membrane and 68 L·m^−2^·h^−1^ for A-0.3-60 after 60 min of surface water ultrafiltration. Smaller flux decrease of the modified membrane is attributed to higher fouling resistance compared to the reference membrane. A number of factors are responsible for better antifouling performance of the modified membrane: lower water contact angle (higher hydrophilicity) and higher absolute value of negative charge compared to the reference A60 membrane. It is known that NOM consists mainly of humic and fulvic acids which feature negative charge at the studied pH = 7.3–7.2 providing better electrostatic repulsion from negatively charged membrane surface in the case of A-0.3-60 membrane compared to the reference membrane [94].

According to the feed and permeate analysis it was revealed that A-0.3-60 membrane is superior to the reference membrane in rejecting organic carbon and iron which is due to the higher negative charge of the skin layer surface promoting better adsorption of positively charged iron ions and colloid particles and repulsion of negatively charged NOM (fulvic and humic acids) (Table 5). Moreover, the A-0.3-60 membrane slightly better removes turbidity compared to the reference membrane due to the abovementioned considerations (Table 5).

Thus, it was revealed that both antifouling and separation performance of polysulfone ultrafiltration membranes are improved by PASA addition into the coagulation bath in membrane preparation via NIPS. This improvement is due to the change of the structure and properties of the skin layer: water contact angle, zeta potential and pore structure.

## 4. Conclusions

A cheap, easy to perform method of membrane surface modification to enhance antifouling stability in water treatment using an anionic flocculant is proposed. Anionic polymeric flocculant based on acrylamide and sodium acrylate (PASA) produced on commercial scale was used for modification of polysulfone ultrafiltration membranes via addition to the aqueous coagulation bath during membrane preparation by NIPS. The influence of PASA concentration at different coagulation bath temperatures on membrane structure, separation and antifouling performance was studied. It was revealed that PASA addition to the coagulation bath affects the kinetics of phase separation due to the substantial increase of coagulant viscosity which results in suppression of macrovoid formation due to delayed demixing mechanism of phase inversion which is confirmed by large increase of membrane formation time. Incorporation of PASA macromolecules into the membrane matrix was proved by FTIR studies revealing that skin layer is modified to the higher degree at all coagulation bath temperatures compared to the bottom membrane layer. PASA addition to the coagulation bath was found to yield the decrease of membrane permeability due to the formation of denser and thicker skin layer. It was shown that modification by PASA significantly changes the properties of the membrane skin layer: pore size increases, hydrophilicity improves, surface roughness increases and zeta potential changes featuring higher negative values at pH 7–9. The modified PSF membranes obtained using 0.3 wt % PASA in the coagulation bath demonstrated better antifouling stability and separation performance towards natural organic matter in surface water ultrafiltration compared to the reference membrane due to adjustment of physico-chemical properties of the skin layer.

## Figures and Tables

**Table 1:** Composition and temperature of coagulant during membrane preparation.

Membrane	Composition of Coagulant	Coagulant Temperature, °C
	c (PASA), wt %	
A25	0	25
A40	0	40
A60	0	60
A70	0	70
A-0.05-25	0.05	25
A-0.05-40		40
A-0.05-60		60
A-0.05-70		70
A-0.1-25	0.1	25
A-0.1-40		40
A-0.1-60		60
A-0.1-70		70
A-0.2-25	0.2	25
A-0.2-40		40
A-0.2-60		60
A-0.2-70		70
A-0.3-25	0.3	25
A-0.3-40		40
A-0.3-60		60
A-0.3-70		70

**Table 2:** Parameters of surface roughness determined by atomic force microscopy (AFM).

Membrane	Coagulation Bath		R_a_, nm	R_q_, nm
	c (PASA), %	T, °C		
A25	0	25	4.6	5.7
A40	0	40	4.7	5.9
A60	0	60	4.7	6.1
A-0.05-60	0.05	60	11.2	14.1
A-0.1-25	0.1	25	6.2	7.1
A-0.1-40	0.1	40	20.7	26.3
A-0.1-60	0.1	60	27.2	35.2
A-0.3-60	0.3	60	20.9	25.4

**Table 3:** Zeta potential of the membrane skin layer.

Membrane Abbreviation		Zeta Potential, mV			
	pH = 3.2	pH = 4.0	pH = 4.6	pH = 7.0	pH = 9.5
A25	8	0	−28	−58	−65
A-0.3-25	28	0	−18	−88	−95

**Table 4:** Characteristics of feed and permeate upon ultrafiltration of humic acid solution.

Characteristics	Feed 0.001 wt % HA Solution in Tap Water	Permeate	
		A25	A-0.3-25
Color (λ = 400 nm)	0.10	0.01	0.01
pH	8.4	8.4	8.4
TOC, mg∙L^−1^	0.54	0	0
c (Fe), µg∙L^−1^	156	0	0

**Table 5:** Characteristics of feed surface water and permeate.

Characteristics	Feed Surface Water	Permeate	
		A60	A-0.3-60
**Turbidity, NTU**	12.0	0.150	0.108
**pH**	7.3	7.2	7.2
**Color (λ = 400 nm)**	0.128	0.017	0.017
**TOC, mg∙L^−1^**	20.4	7.1	3.8
**c (Fe), µg∙L^−1^**	410	0.70	0.38
